# Supplementary material for: Supercoiled DNA recognition and cleavage control in topoisomerase VI
Source: Nat Commun. 2026 Feb 16;17:3092. doi: 10.1038/s41467-026-69491-0 (PMC13040014; doi:10.1038/s41467-026-69491-0)
Supplement: Supplementary file 1 — Supplementary Information [file 41467_2026_69491_MOESM1_ESM.pdf]

Supplementary Information

Supercoiled DNA recognition and cleavage control in topoisomerase VI

Daniel E. Richman [1], Timothy J. Wendorff [1]#, Fahad Rashid [1], Curtis Beck [1], Qianyun Yan [1], Haley R. Johnson [2,3], Ryan A. Eckerty [3], Jonathan M. Fogg [3,4], Matthew L. Baker [5], Lynn Zechiedrich [2,3,4], James M. Berger [1]*

[1] Department of Biophysics & Biophysical Chemistry, Johns Hopkins University School of Medicine, Baltimore, MD USA
[2] Graduate Program in Quantitative & Computational Biosciences, Baylor College of Medicine, Houston, TX, USA
[3] Department of Molecular Virology & Microbiology, Baylor College of Medicine, Houston, TX USA
[4] Verna and Marrs McLean Department of Biochemistry & Pharmacology, Baylor College of Medicine, Houston, TX, USA
[5] Department of Biochemistry & Molecular Biology, UTHealth Houston, Houston, TX, USA

#Current affiliation: Genentech, Inc., South San Francisco, CA, USA
*Correspondence to James M. Berger, jmberger@jhmi.edu

Contents

Supplementary Fig. 1: Minicircle relaxation by WT Top6
Supplementary Fig. 2: Summary of the cryoEM processing workflow for the WT Top6 dataset
Supplementary Fig. 3: CryoEM diagnostics for WT Top6•mcDNA•ADPNP symmetric state reconstruction
Supplementary Fig. 4: B-subunit 'straps' and comparison of ATP region with MutL
Supplementary Fig. 5: Top6A structures in different studies and Top6A interactions with Top6B and DNA
Supplementary Fig. 6: Conservation and function of the WH-TOPRIM latch
Supplementary Fig. 7: Conservation of the electrostatic clasp and optimization of cleavage activity
Supplementary Fig. 8: Summary of the cryoEM processing workflow for the Top6$^{(A:E342Q)}$ dataset
Supplementary Fig. 9: CryoEM diagnostics for Top6$^{(A:E342Q)}$•mcDNA•ADPNP symmetric state reconstruction
Supplementary Fig. 10: Top6A$^{(E342Q)}$-DNA interactions and properties of the cleaved DNA sequence
Supplementary Fig. 11: CryoEM diagnostics and structural comparisons of the asymmetric states
Supplementary Fig. 12: Correlation between DNA bending and protein domain movements
Supplementary Fig. 13: Comparisons of TOPRIM domain-DNA interactions in related systems
Supplementary Table 1: Model and map validation parameters
Supplementary Table 2: Purine (R)/pyrimidine (Y) assignment derived from density map of mcDNA
Supplementary Table 3: Displacements and rotations of Top6 domains
Supplementary Table 4: Angles of DNA deviation away from horizontal at the central kink
Supplementary References

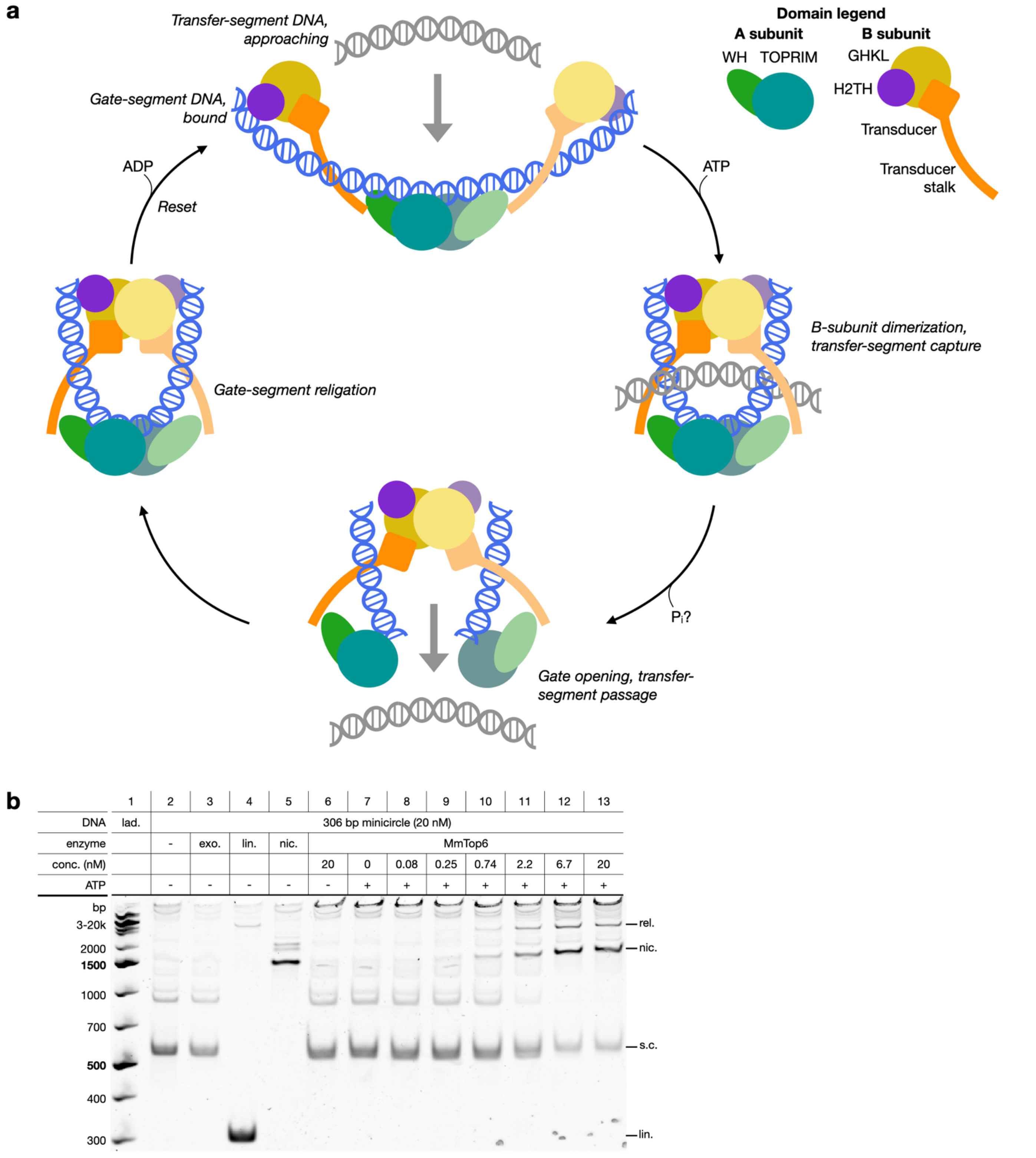

Supplementary Fig. 1: Strand passage reaction and minicircle relaxation by WT *M. mazei* Top6
a Schematic illustration of the Top6 strand passage reaction. For simplicity, the B-subunit CTD is not shown.
b Native agarose gel titrating different amounts of Top6 vs. 20 nM mcDNA to assess supercoil relaxation. Abbreviations: lad.: Thermo Scientific GeneRuler 1 kb Plus ladder, rel.: relaxed, exo.: T5 exonuclease-treated mcDNA control, lin.: EcoRV-linearized mcDNA control, nic.: Nt.BstNBI-nicked mcDNA control. The result shown is from 1 of 4 distinct experiments. An uncropped gel image is provided as a Source Data file.

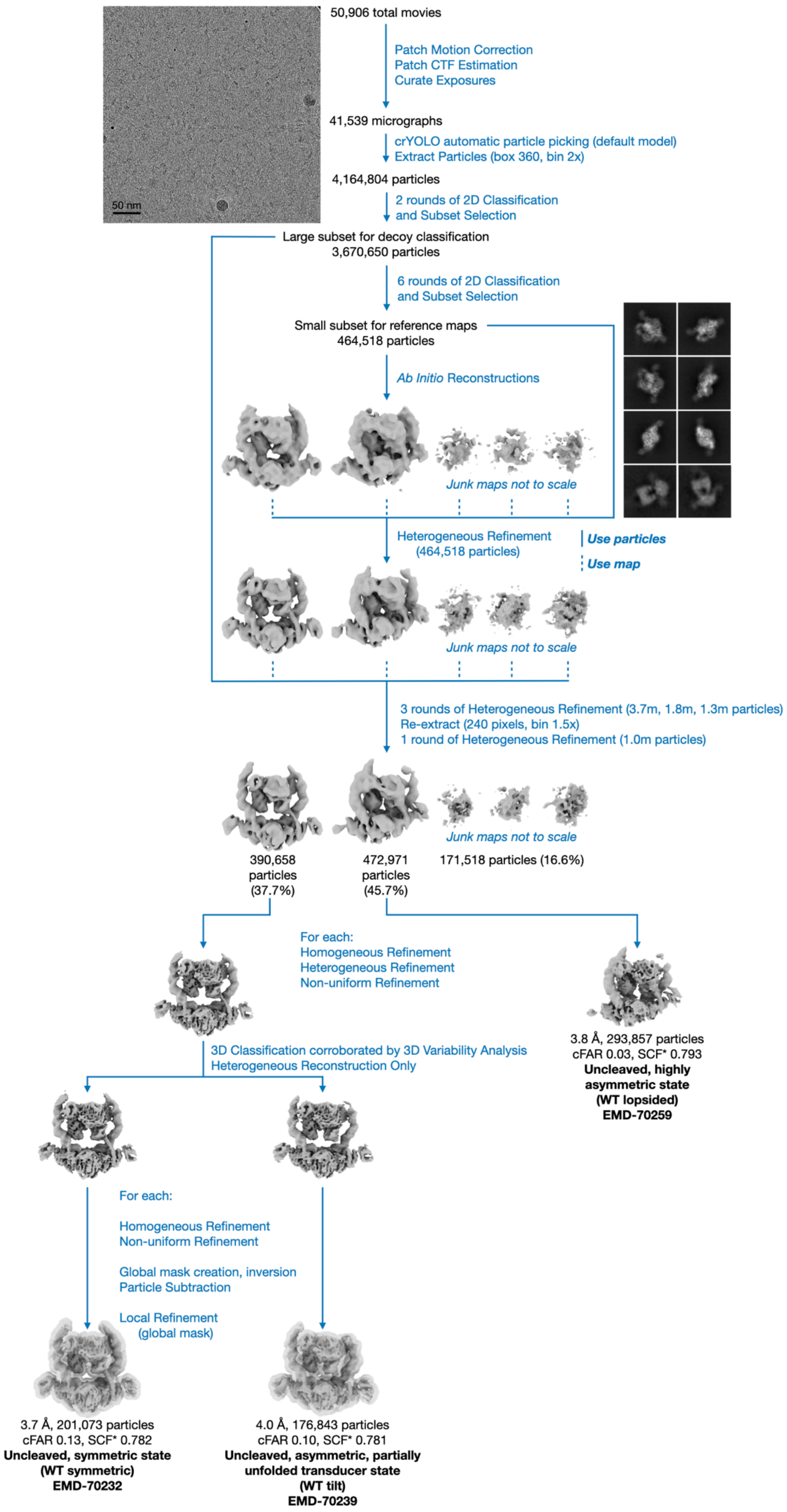
50 nm
50,906 total movies
Patch Motion Correction
Patch CTF Estimation
Curate Exposures
41,539 micrographs
crYOLO automatic particle picking (default model)
Extract Particles (box 360, bin 2x)
4,164,804 particles
2 rounds of 2D Classification
and Subset Selection
Large subset for decoy classification
3,670,650 particles
6 rounds of 2D Classification
and Subset Selection
Small subset for reference maps
464,518 particles
Ab Initio Reconstructions
Junk maps not to scale
Heterogeneous Refinement
(464,518 particles)
Use particles
Use map
Junk maps not to scale
3 rounds of Heterogeneous Refinement (3.7m, 1.8m, 1.3m particles)
Re-extract (240 pixels, bin 1.5x)
1 round of Heterogeneous Refinement (1.0m particles)
Junk maps not to scale
390,658
particles
(37.7%)
472,971
particles
(45.7%)
171,518 particles (16.6%)
For each:
Homogeneous Refinement
Heterogeneous Refinement
Non-uniform Refinement
3.8 Å, 293,857 particles
cFAR 0.03, SCF* 0.793
Uncleaved, highly asymmetric state (WT lopsided)
EMD-70259
3D Classification corroborated by 3D Variability Analysis
Heterogeneous Reconstruction Only
For each:
Homogeneous Refinement
Non-uniform Refinement
Global mask creation, inversion
Particle Subtraction
Local Refinement
(global mask)
3.7 Å, 201,073 particles
cFAR 0.13, SCF* 0.782
Uncleaved, symmetric state (WT symmetric)
EMD-70232
4.0 Å, 176,843 particles
cFAR 0.10, SCF* 0.781
Uncleaved, asymmetric, partially unfolded transducer state (WT tilt)
EMD-70239

Supplementary Fig. 2: Summary of the cryoEM processing workflow for the WT Top6 dataset
Processes are denoted with blue arrows and labeled with CryoSPARC[1–3] job names, except for automatic particle picking, which was performed using crYOLO[4]. Data objects (movies, micrographs, particles, maps) are described with black text or grayscale maps. For 2D Classification jobs, the following custom parameters were used: 200 or 100 classes, 30 O-EM iterations, 2 full iterations, batch size per class 400. For *ab initio* reconstructions, acceptable maps were chosen from two separate jobs containing 3 and 6 classes, respectively, and junk reference maps were selected from an additional job that was aborted after initialization. Homogeneous and non-uniform refinement jobs used the following custom parameters: minimize over per-particle scale, optimize per-group CTF parameters, 2 classes, O-EM learning rate 0.75, half-life 75%; class similarity 0.25, per-particle scale from input data. Mask output *via* the Volume Tools job used an input map, threshold 0.15, dilate 2, pad 6. Inversion of the mask used an input mask, dilate 1, pad 1. Local Refinement jobs used the global mask and the inverse-mask subtracted particles and the following custom parameters: use pose/shift Gaussian prior, re-center rotations and shifts each iteration.

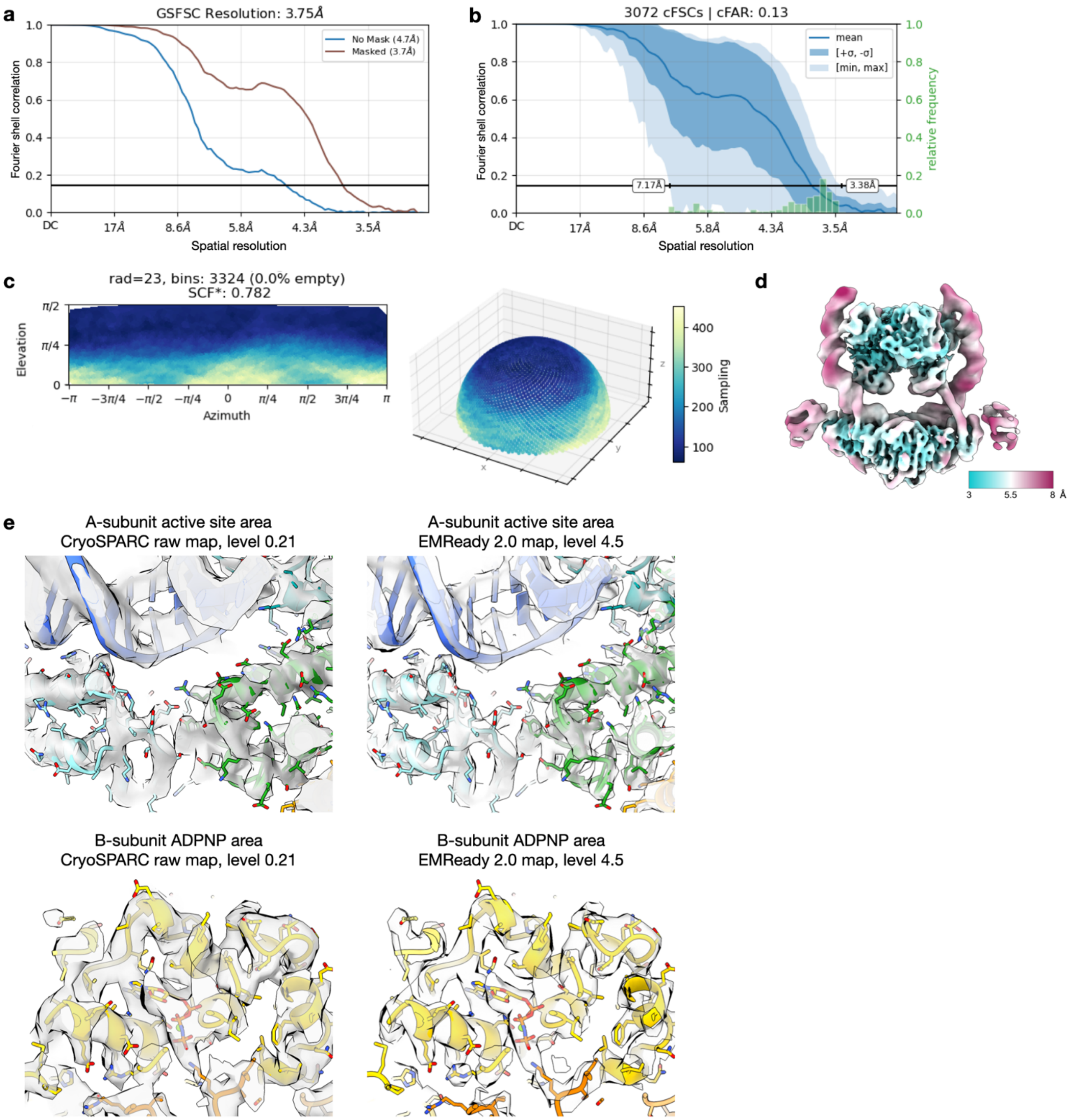

Supplementary Fig. 3: CryoEM diagnostics for the WT Top6•mcDNA•ADPNP symmetric state reconstruction (EMD-70232)

Plots **a**–**c** adapted from CryoSPARC Orientation Diagnostics[5,6]. **a** Fourier shell correlation (FSC) plot. The horizontal black line marks the 0.143 gold-standard criterion and the gold-standard FSC (GSFSC) value of the masked density is reported above of the plot. **b** Statistical summary of 3072 conical Fourier shell correlations (cFSCs) versus spatial resolution and histogram representing the proportion (relative frequency) of the cFSCs crossing the 0.143 threshold at each resolution. The cFSC area ratio (cFAR) is reported above the plot. **c** Fourier sampling visualized with an azimuth and elevation parameterization (left) and on a hemisphere in Fourier space (right). Note the sampling is antipodally symmetric, so only elevation > 0 is shown. The sampling compensation factor (SCF*) and its radius and bin parameters are reported above the azimuth and elevation plot. **d** Local resolution[7] displayed on the locally filtered density with heatmap coloring. **e** Model-in-map fits of representative areas.

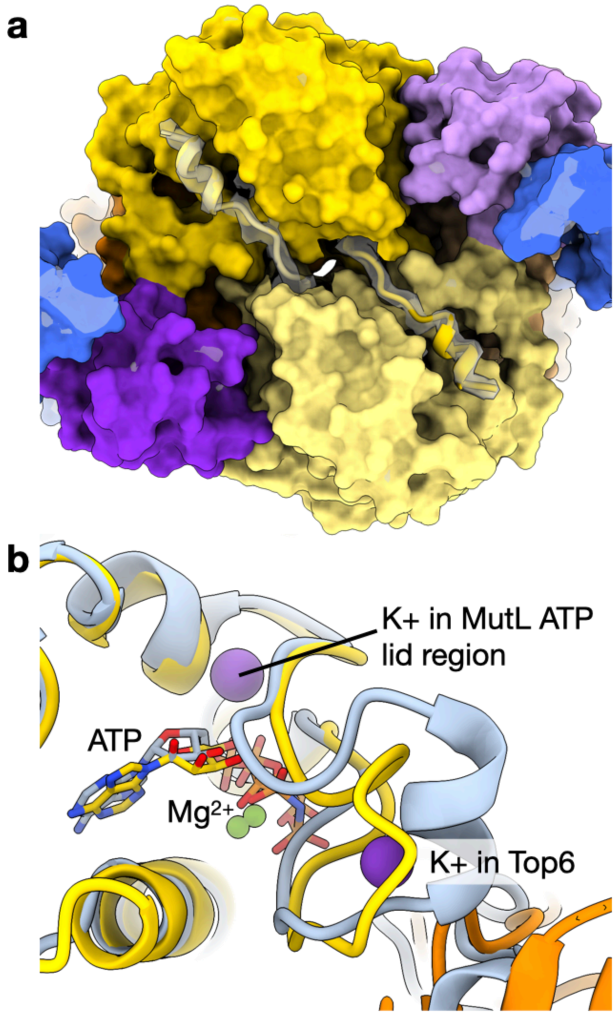

Supplementary Fig. 4: B-subunit straps and comparison of ATP region with MutL
a Surface representation of the Top6-mcDNA model in top-down view with the N-terminal straps of the B subunits depicted as cartoons. The cryoEM density of the straps is shown as semitransparent gray. **b** Superposition of the ATP-binding regions of the GHKL domains of MutL[8] (PDB ID: 1NHI) and Top6 showing the locations of associated potassium ions (purple spheres). The MutL GHKL domain is colored light blue, the Top6 GHKL and transducer domains are yellow and orange (respectively), magnesium ions are light green, and ATP molecules are color-matched to their respective GHKL domains along with conventional elemental coloring.

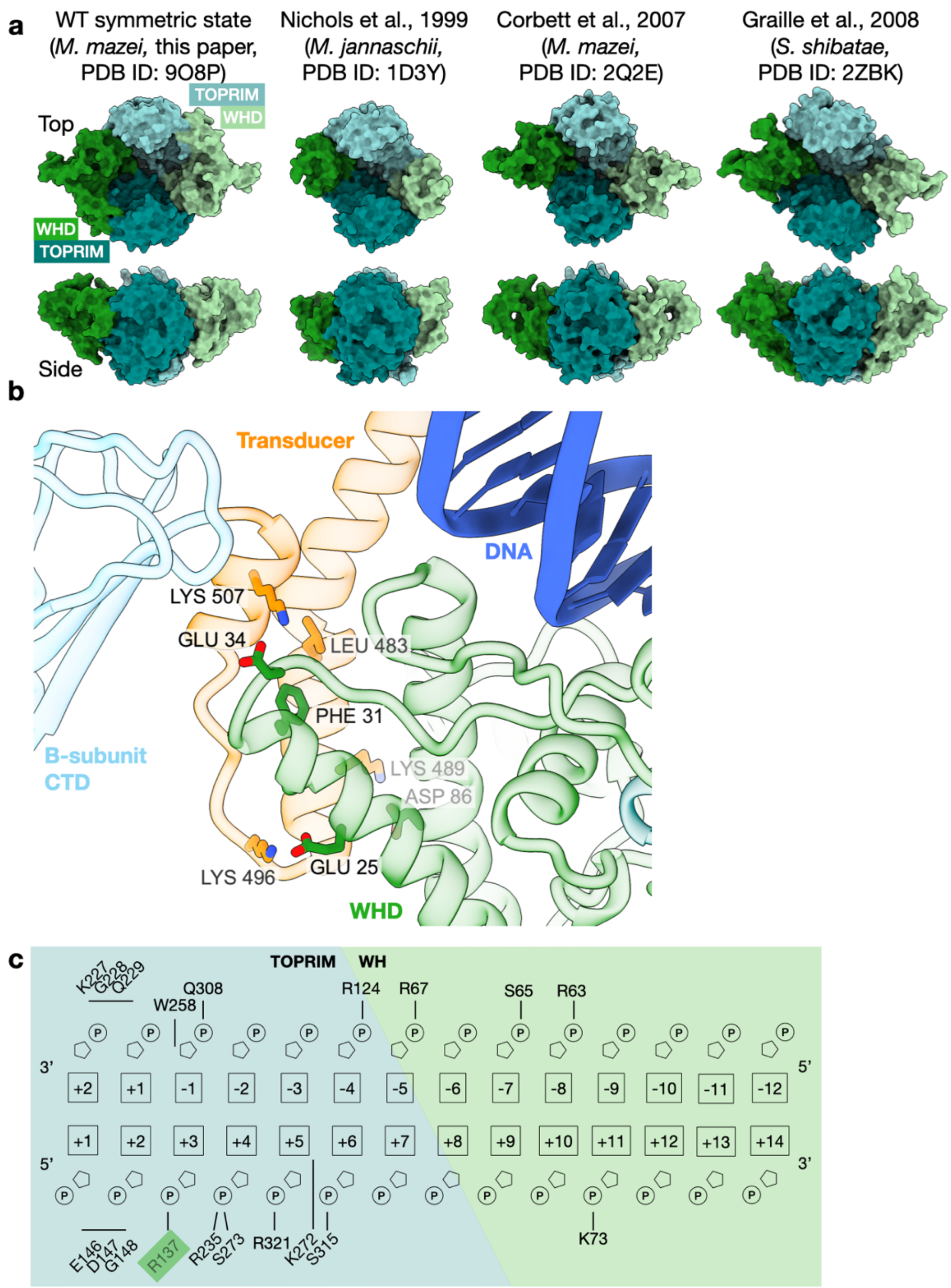

Supplementary Fig. 5: Top6A structures in different studies and Top6A interactions with Top6B and DNA
a Comparison of the organization of TOPRIM and WH domains in Top6A dimers from Top6•mcDNA•ADPNP (symmetric state) in this paper and three structures without DNA from the literature[9–11]. **b** Close-up of the interface between the WH domain and the hairpin of the transducer stalk shown in cartoon representation. Interfacial side chains are shown as sticks. **c** Schematic of the protein-DNA contacts in the Top6A channel. Only one set of Top6A monomer contacts are shown for clarity.

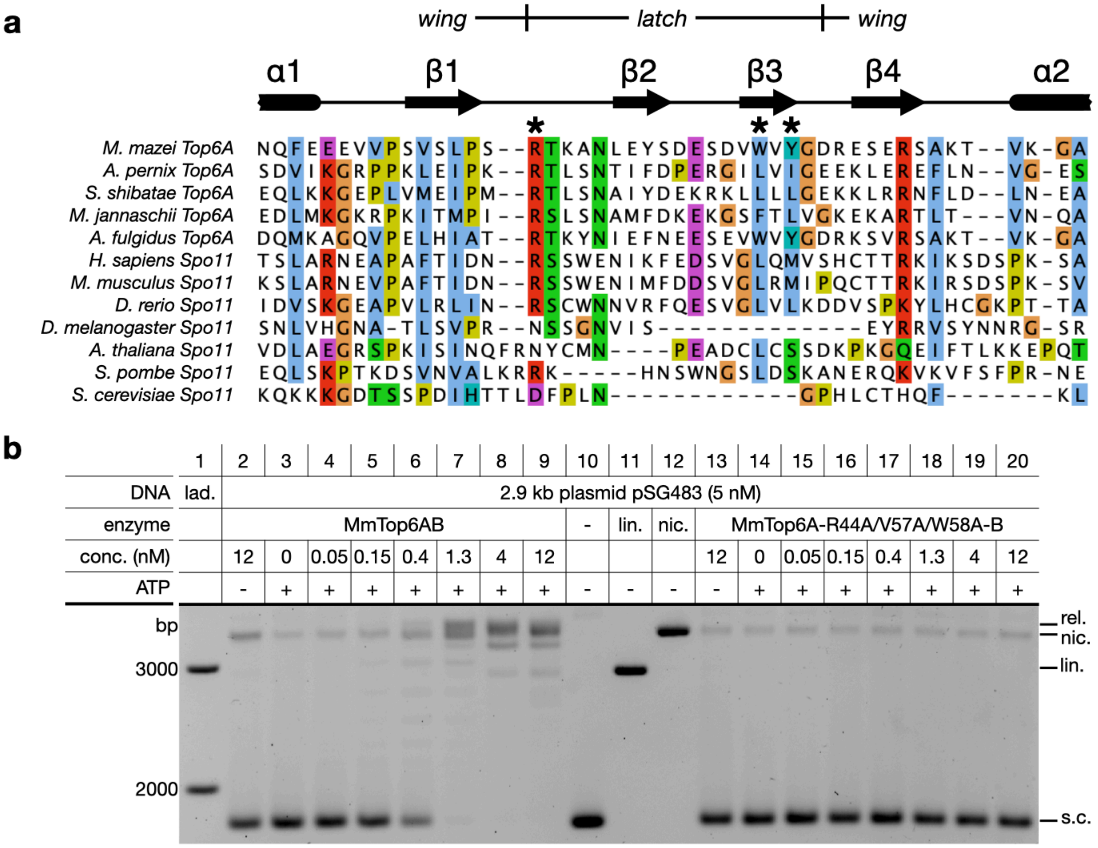

Supplementary Fig. 6: Conservation and functional importance of the WH-TOPRIM latch

a Alignment of Top6A and Spo11 WHD sequences from several representative species. Asterisks denote the positions of R44, V57, and W58 in *M. mazei* Top6A. **b** Native agarose gel comparing negative supercoil relaxation by the Top6$^{(A:R44A/V57A/W58A)}$ latch variant with WT Top6. The migration positions of supercoiled (s.c.), linearized (lin.), nicked (nic.), and relaxed (rel.) plasmid species are labeled. The result shown is from 1 of 2 virtually identical experimental replicates. An uncropped gel image is provided as a Source Data file.

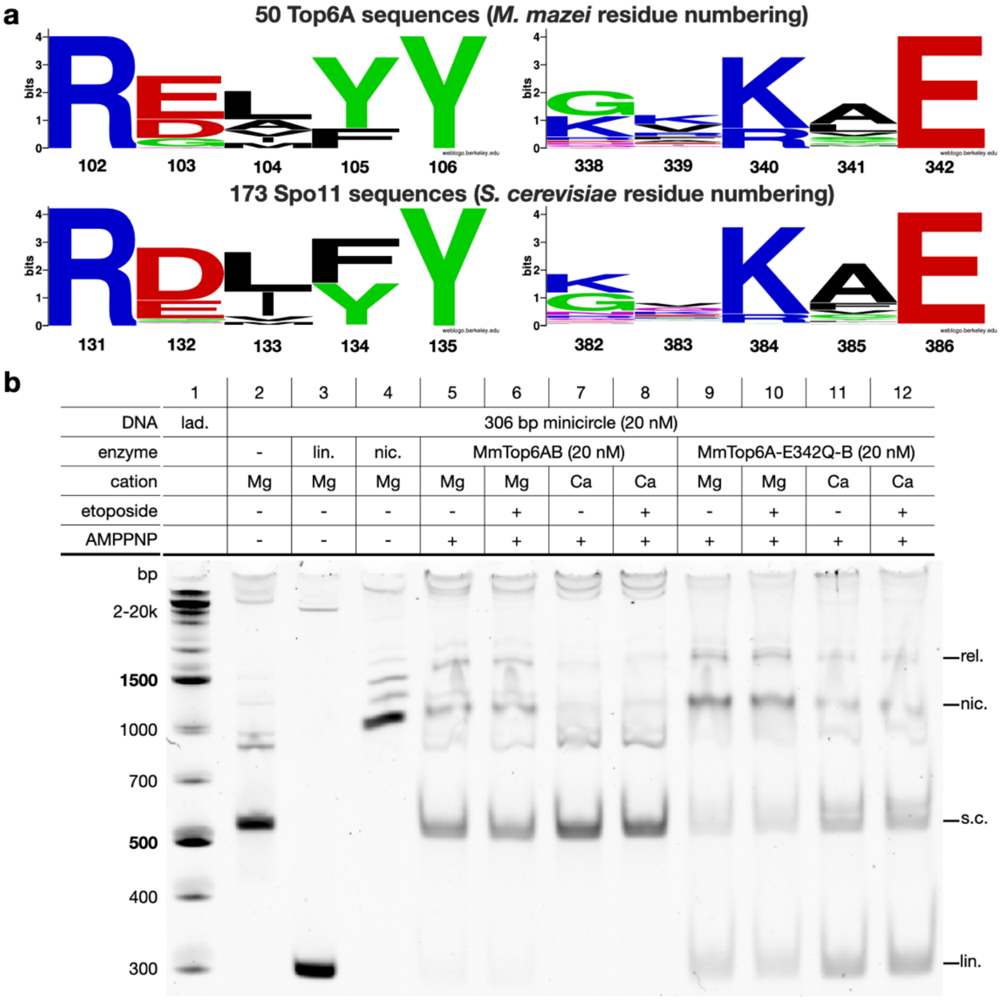

Supplementary Fig. 7: Conservation of the electrostatic clasp and optimization of DNA cleavage by Top6
a WebLogo[12] plots highlighting the conservation of R102 and E342 (as well as the DNA-linking tyrosine, Y106) across 50 Top6 and 173 Spo11 species (as curated by Allen and Maxwell, 2024[13]). **b** Native agarose gel assessing mcDNA cleavage by WT Top6 and Top6$^{(A:E342Q)}$ in the presence or absence of magnesium, calcium, or etoposide. Abbreviations: lad.: Thermo Scientific GeneRuler 1 kb Plus ladder, lin.: EcoRV-linearized mcDNA control, nic.: Nt.BstNBI-nicked mcDNA control. The result shown is from 1 of 2 virtually identical experimental replicates. An uncropped gel image is provided as a Source Data file.

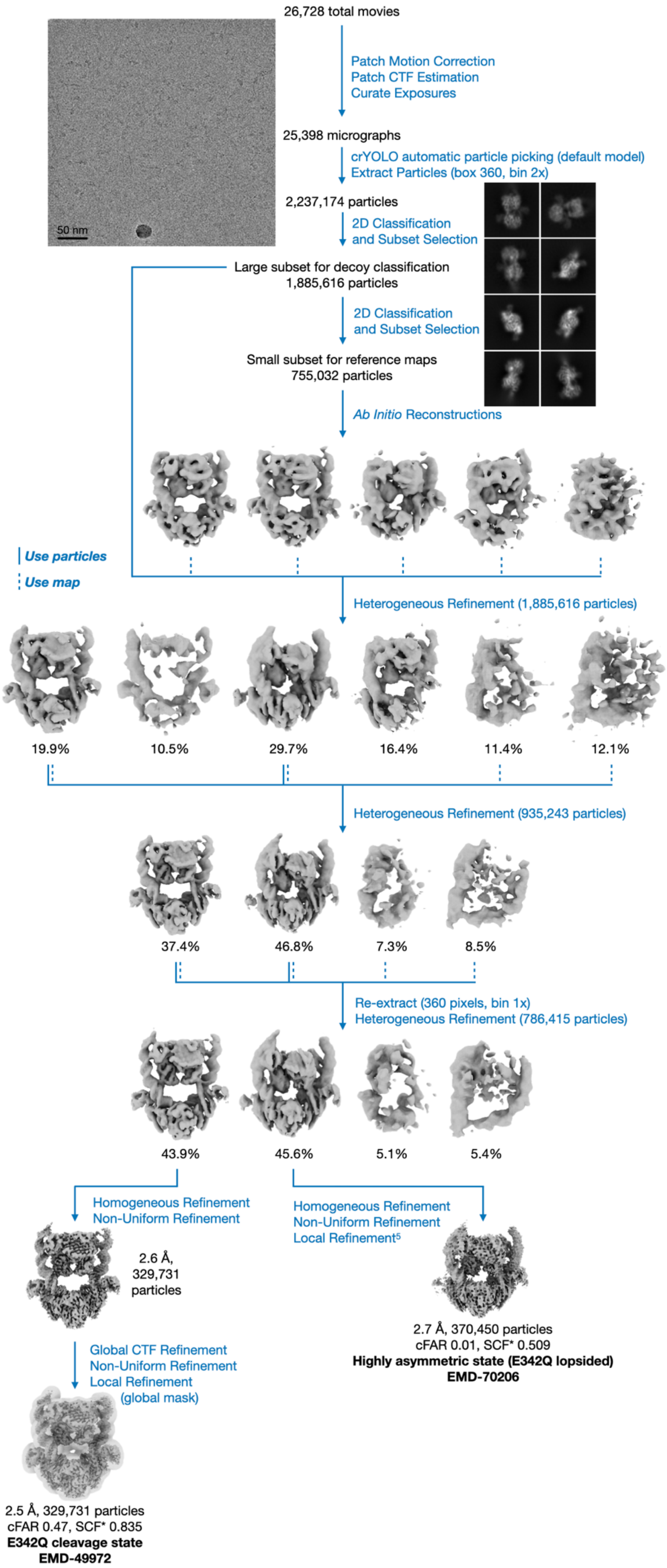
26,728 total movies
Patch Motion Correction
Patch CTF Estimation
Curate Exposures
25,398 micrographs
crYOLO automatic particle picking (default model)
Extract Particles (box 360, bin 2x)
2,237,174 particles
50 nm
2D Classification
and Subset Selection
Large subset for decoy classification
1,885,616 particles
2D Classification
and Subset Selection
Small subset for reference maps
755,032 particles
Ab Initio Reconstructions
Use particles
Use map
Heterogeneous Refinement (1,885,616 particles)
19.9%
10.5%
29.7%
16.4%
11.4%
12.1%
Heterogeneous Refinement (935,243 particles)
37.4%
46.8%
7.3%
8.5%
Re-extract (360 pixels, bin 1x)
Heterogeneous Refinement (786,415 particles)
43.9%
45.6%
5.1%
5.4%
Homogeneous Refinement
Non-Uniform Refinement
2.6 Å,
329,731
particles
Homogeneous Refinement
Non-Uniform Refinement
Local Refinement5
2.7 Å, 370,450 particles
cFAR 0.01, SCF* 0.509
Highly asymmetric state (E342Q lopsided)
EMD-70206
Global CTF Refinement
Non-Uniform Refinement
Local Refinement
(global mask)
2.5 Å, 329,731 particles
cFAR 0.47, SCF* 0.835
E342Q cleavage state
EMD-49972

Supplementary Fig. 8: Summary of the cryoEM processing workflow for the Top6$^{(A:E342Q)}$ dataset

CryoSPARC job names, crYOLO picking, and data objects are denoted as in Supplementary Fig. 2. For 2D Classification jobs, the following custom parameters were used: 100 classes, 30 O-EM iterations, 2 full iterations, batch size per class 400. For *Ab Initio* Reconstruction, acceptable and junk maps were chosen from four different jobs run with 2, 3, 4, and 5 classes, respectively. Global CTF Refinement used the following custom parameters: fit spherical aberration, tetrafoil, anisotropic magnification; 2 iterations. Non-uniform Refinement used the custom parameter minimize over per particle scale. Local Refinement was run with a global mask and the following custom parameters: use pose/shift Gaussian prior, re-center rotations and shifts, initial lowpass resolution 5 Å.

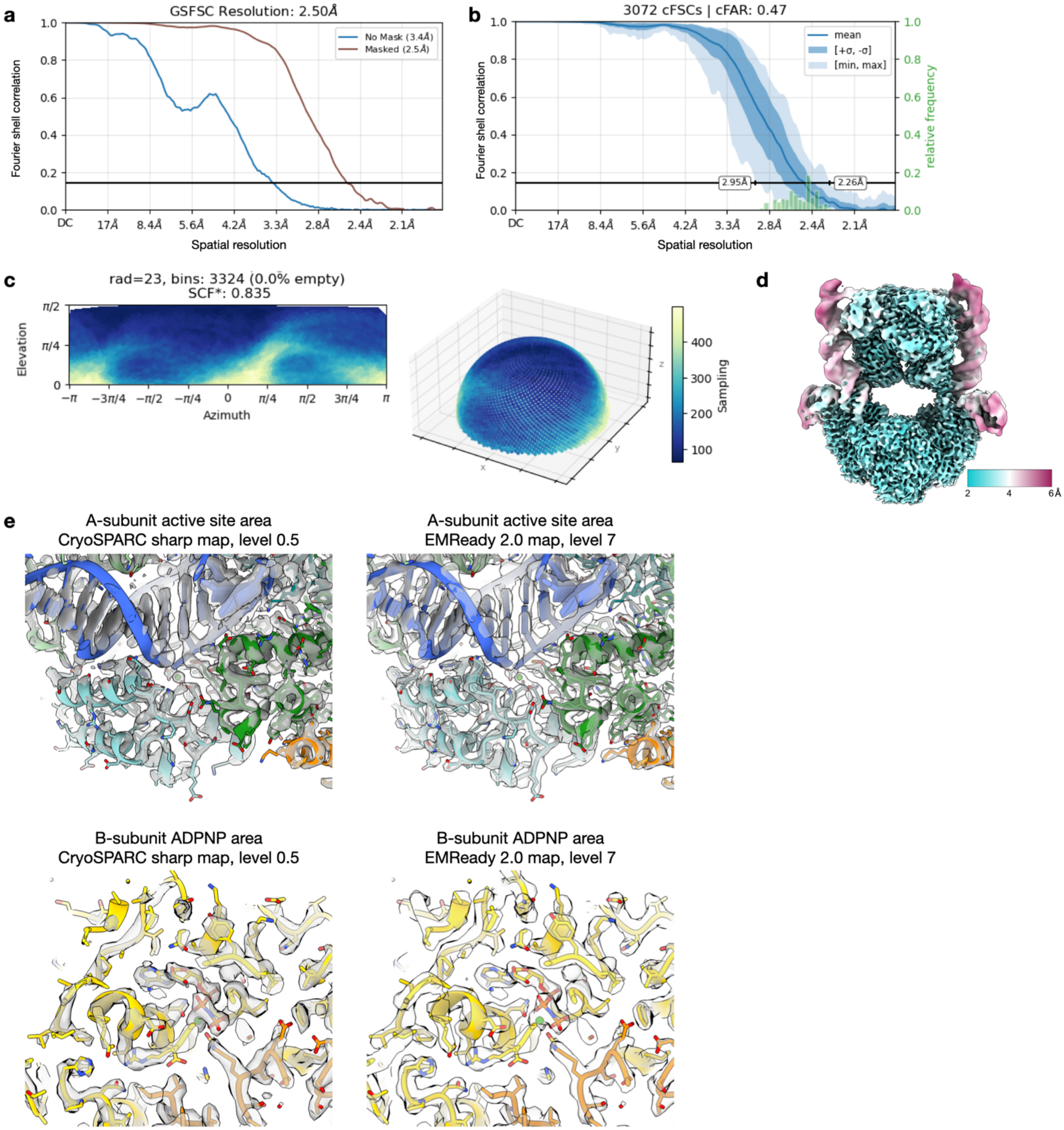

Supplementary Fig. 9: CryoEM diagnostics for the Top6$^{(A:E342Q)}$•mcDNA•ADPNP symmetric state reconstruction (EMD-49972)

Plots are displayed and annotated as in Supplementary Fig. 3.

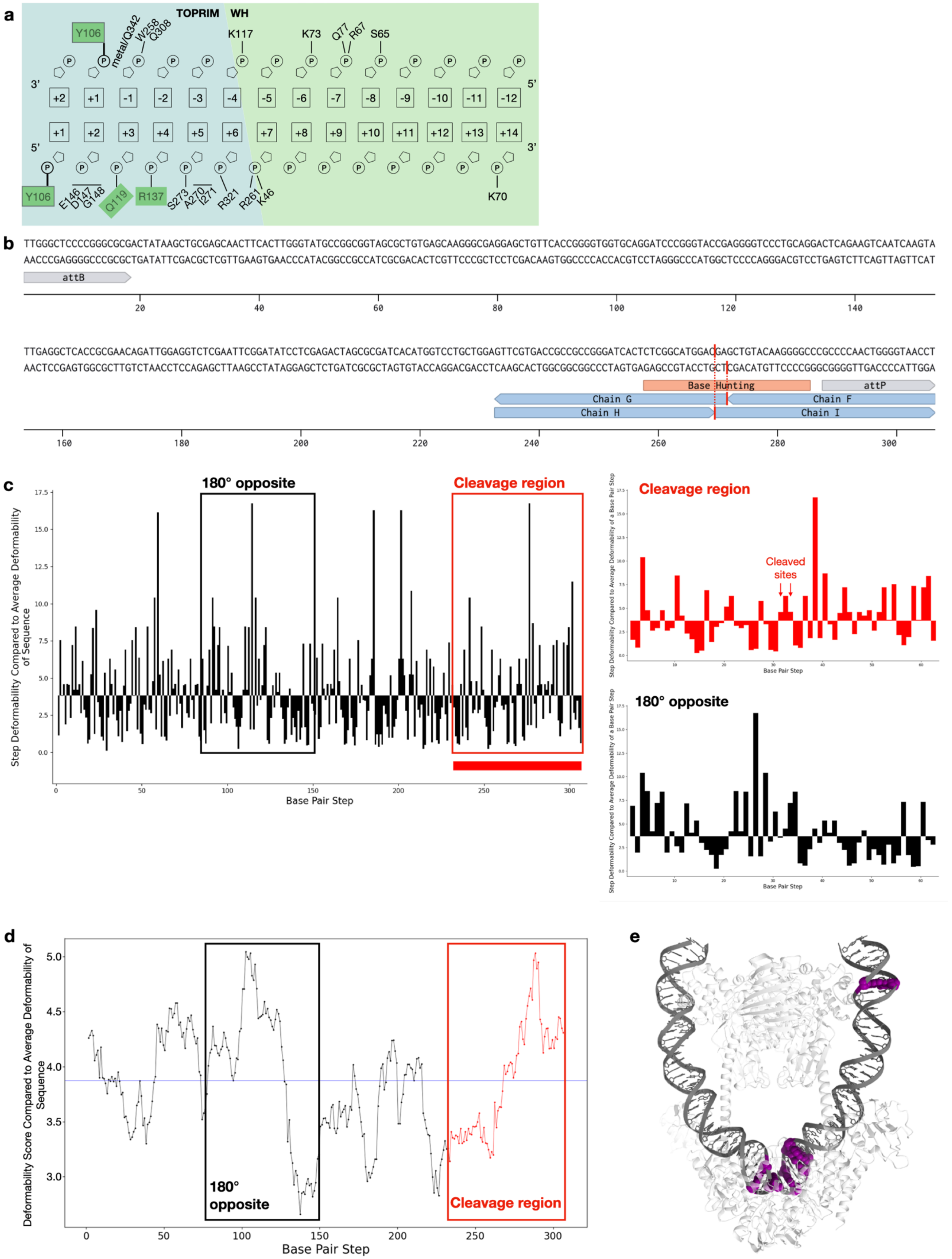

a
TOPRIM
WH
Y106
metal/Q342
W258
Q308
K117
K73
Q77
R67
S65
3'
5'
+2
+1
-1
-2
-3
-4
-5
-6
-7
-8
-9
-10
-11
-12
+3
+4
+5
+6
+7
+8
+9
+10
+11
+12
+13
+14
E146
D147
G148
Q119
R137
S273
A270
I271
R321
R261
K46
K70
b
attB
Base Hunting
attP
Chain G
Chain F
Chain H
Chain I
c
180° opposite
Cleavage region
Step Deformability Compared to Average Deformability of Sequence
Base Pair Step
Cleaved sites
Step Deformability Compared to Average Deformability of a Base Pair Step
d
Deformability Score Compared to Average Deformability of Sequence
180° opposite
Cleavage region
Base Pair Step
e

Supplementary Fig. 10: Top6A$^{(E342Q)}$-DNA interactions and deformability properties of the cleaved DNA sequence

a Schematic of the protein-DNA contacts observed in the Top6A$^{(E342Q)}$ channel. Only one monomer is shown for clarity. **b** Base pair sequence of the minicircle DNA. The locations of the cleavage region used in the Base Hunting algorithm, the DNA chain names in the cleavage-state model (PDB ID: 9O0G), and the recombinase sites attB/P are annotated. The cleavage locations are marked with red, vertical lines. Sequence data are included as a Source Data file. **c** Plot of the sequence-dependent deformability for individual steps of the 306 bp minicircle, with insets featuring the cleavage region and the region on the opposite side of the minicircle. Numerical data are included as a Source Data file. **d** Plot of sequence-dependent deformability score averaged within a sliding window of 30 bp to condense and increase the legibility of trends in the data of panel c. Connecting lines between points are used to guide the eye. The blue horizontal line (3.9 degrees^3•Å^3) represents average deformability. Numerical data are included as a Source Data file. **e** Structure of Top6$^{(A:E342Q)}$-DNA cleavage complex depicted in cartoon rendering and showing the positions of A-form base pair steps DNA (protein in light grey, DNA in dark grey, A-form like region in purple). DNA from the Top6$^{(A:E342Q)}$ cleavage structure was analyzed using X3DNA-DSSR[14].

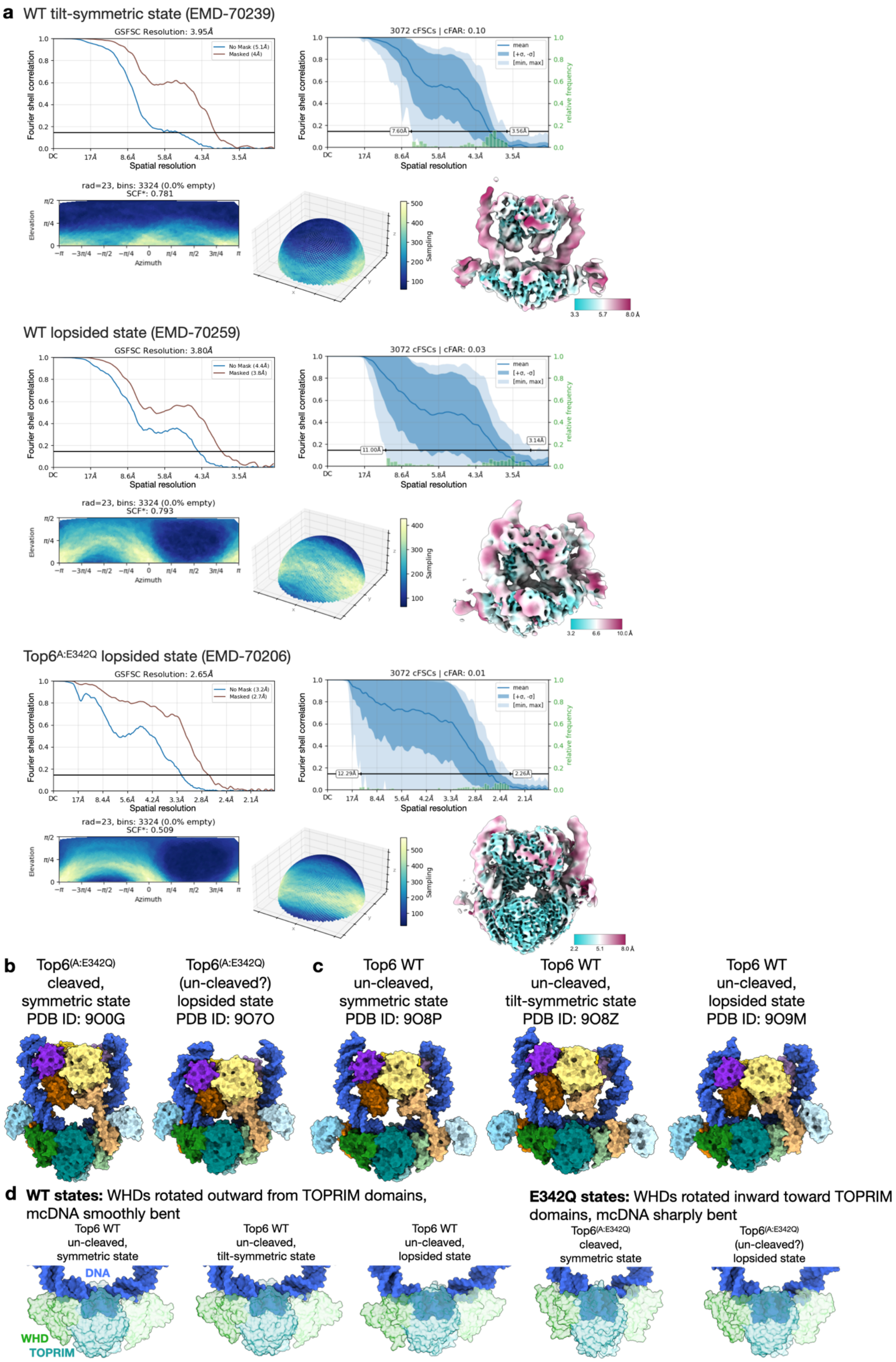
a
WT tilt-symmetric state (EMD-70239)
GSFSC Resolution: 3.95Å
No Mask (5.1Å)
Masked (4Å)
3072 cFSCs | cFAR: 0.10
7.60Å
3.56Å
Fourier shell correlation
relative frequency
Spatial resolution
rad=23, bins: 3324 (0.0% empty)
SCF*: 0.781
Elevation
Azimuth
Sampling
WT lopsided state (EMD-70259)
GSFSC Resolution: 3.80Å
No Mask (4.4Å)
Masked (3.8Å)
3072 cFSCs | cFAR: 0.03
11.00Å
3.14Å
rad=23, bins: 3324 (0.0% empty)
SCF*: 0.793
Top6^(A:E342Q) lopsided state (EMD-70206)
GSFSC Resolution: 2.65Å
No Mask (3.2Å)
Masked (2.7Å)
3072 cFSCs | cFAR: 0.01
12.29Å
2.26Å
rad=23, bins: 3324 (0.0% empty)
SCF*: 0.509
b
Top6(A:E342Q) cleaved, symmetric state PDB ID: 9O0G
Top6(A:E342Q) (un-cleaved?) lopsided state PDB ID: 9O7O
c
Top6 WT un-cleaved, symmetric state PDB ID: 9O8P
Top6 WT un-cleaved, tilt-symmetric state PDB ID: 9O8Z
Top6 WT un-cleaved, lopsided state PDB ID: 9O9M
d
WT states: WHDs rotated outward from TOPRIM domains, mcDNA smoothly bent
Top6 WT un-cleaved, symmetric state
Top6 WT un-cleaved, tilt-symmetric state
Top6 WT un-cleaved, lopsided state
E342Q states: WHDs rotated inward toward TOPRIM domains, mcDNA sharply bent
Top6(A:E342Q) cleaved, symmetric state
Top6(A:E342Q) (un-cleaved?) lopsided state
DNA
WHD
TOPRIM

Supplementary Fig. 11: CryoEM diagnostics and structural comparisons of the asymmetric states

a Diagnostic plots for the asymmetric states reconstructed from the WT Top6 and Top6$^{(A:E342Q)}$ datasets (WT tilt-symmetric: 9O8Z/EMD-70239, WT lopsided: 9O9M/EMD-70259, Top6$^{(A:E342Q)}$ lopsided: 9O7O/EMD-70206. Plots are displayed and annotated as in Supplementary Fig. 3. **b,c** Surface representations of all Top6$^{(A:E342Q)}$ and WT Top6 models, respectively (Top6$^{(A:E342Q)}$ cleavage: 9O0G/EMD-49972), Top6$^{(A:E342Q)}$ lopsided: 9O7O/EMD-70206; WT symmetric: 9O8P/EMD-70232, WT tilt-symmetric: 9O8Z/EMD-70239, WT lopsided: 9O9M/EMD-70259). Domains are colored as per Fig. 1. **d** Surface representations comparing the WHD (light green) positions and DNA (dark blue) bends among all states.

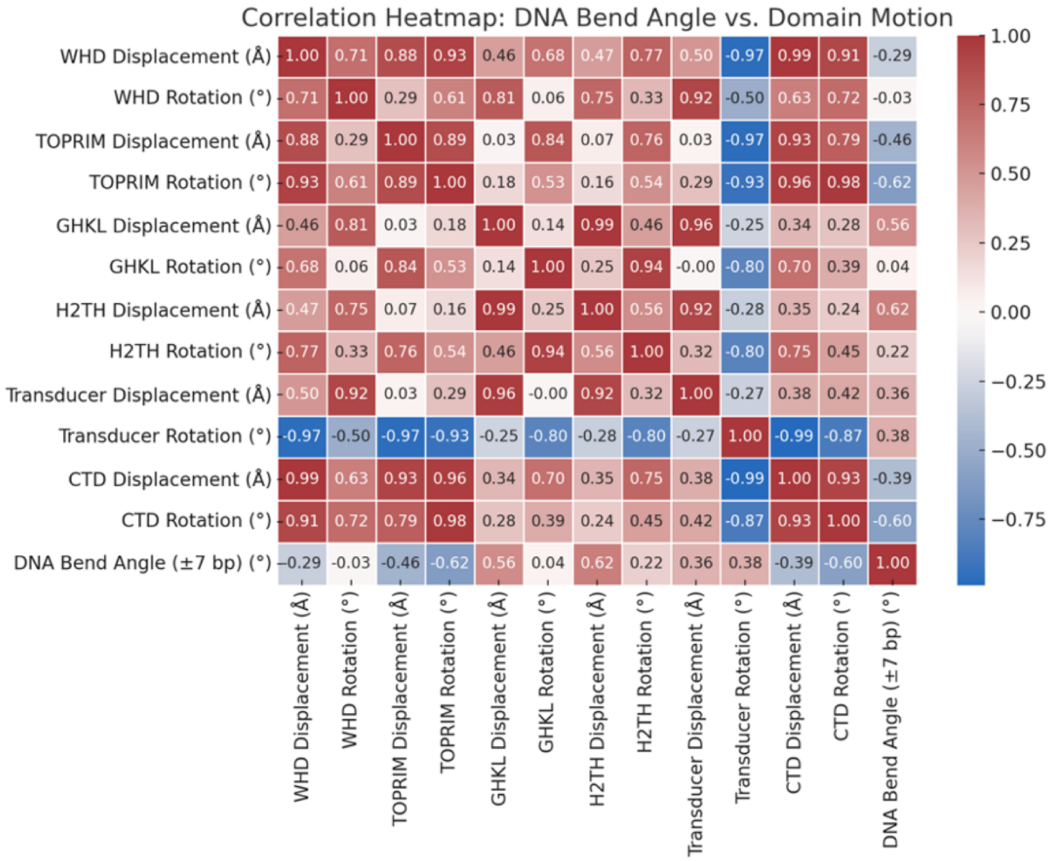

Supplementary Fig. 12: Correlation between DNA bending and protein domain movements

Heatmap showing Pearson correlation coefficients between DNA bend angle (±7 base pairs around a fixed cleavage-centered inflection point) and the translational and rotational motions of the six structurally defined Top6 domains. Motion was quantified as either the center-of-mass displacement or principal axis rotation relative to the Top6$^{(A:E342Q)}$ symmetric state structure. Positive correlations (blue) indicate increased motion with greater DNA bending; negative correlations (red) reflect reduced movement in highly bent states.

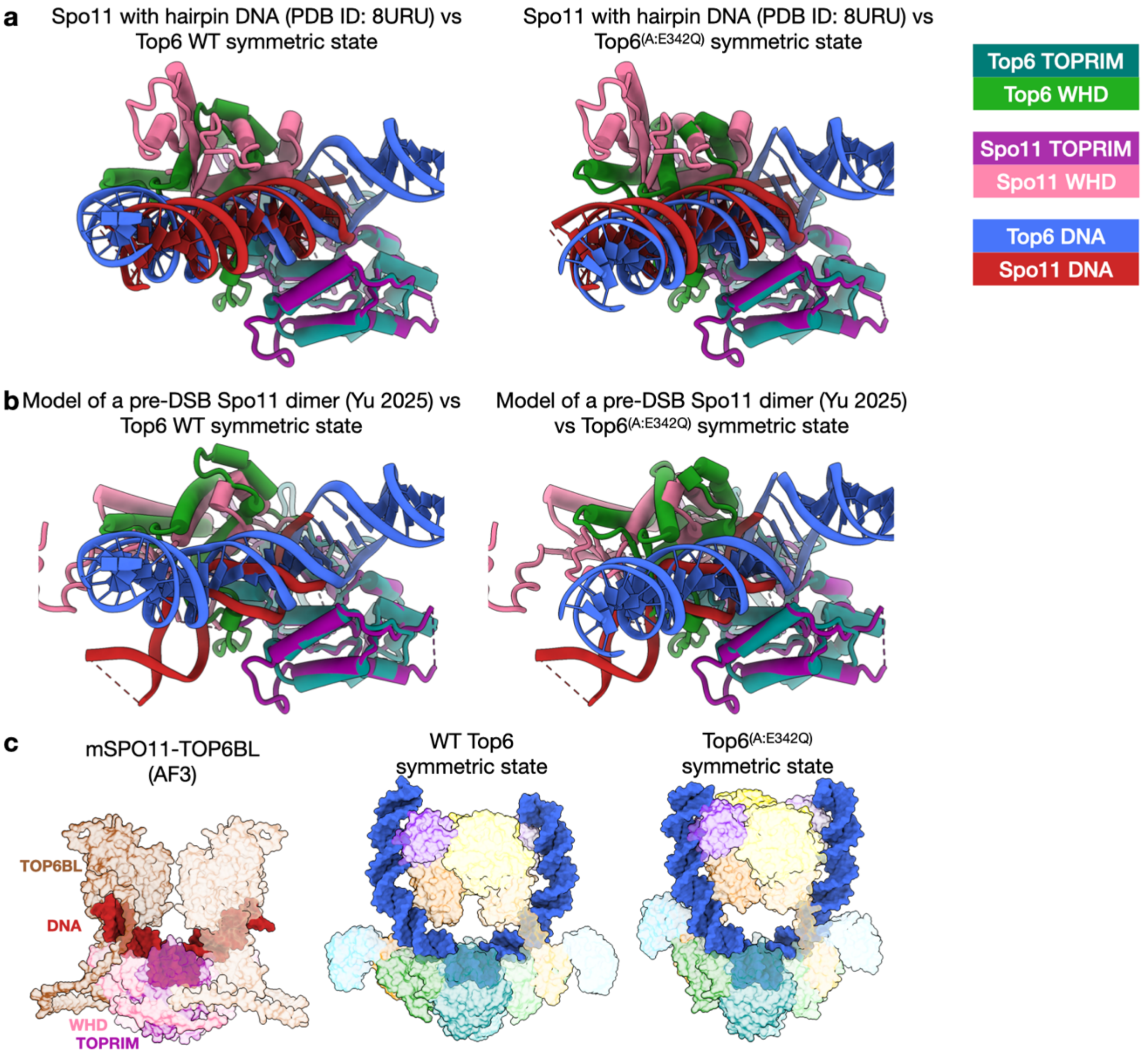

d *Gray cells duplicate Extended Data Fig. 4c, Zheng 2025*

**mSPO11** (Zheng 2025, AF3)	**ySpo11** (Yu 2025, PDB ID: 8URU)	**Top6** (this paper, PDB ID: 9O8P)
K106	K104	
R134	R131	
Y138	Y135	
K175	K173	E146, D147, G148
Y281	Y292	W258
H334	R344	Q308
K110		R124
R345		R321
	R266	R235

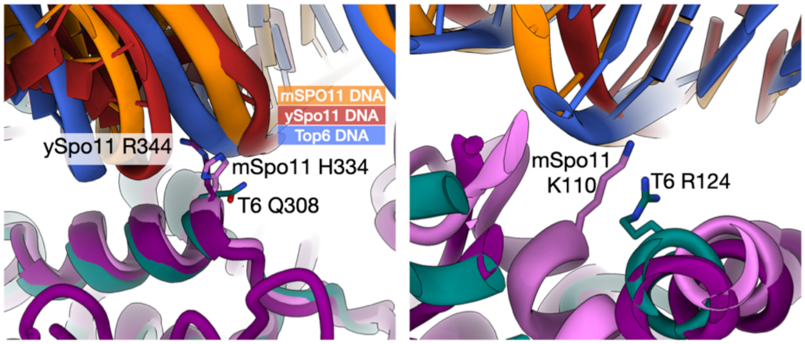

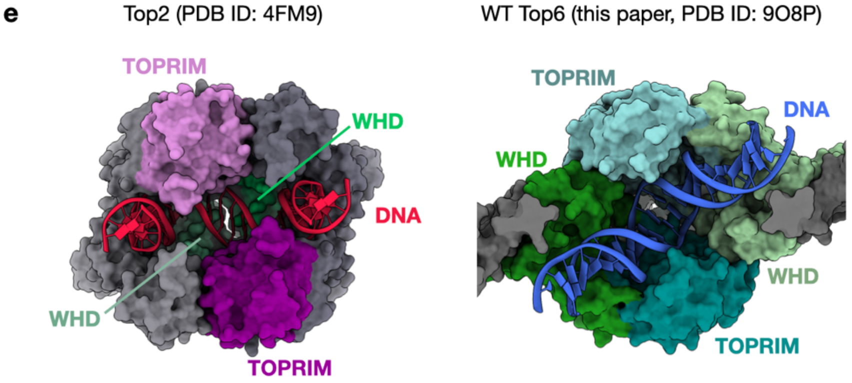

Supplementary Fig. 13: Comparisons of TOPRIM domain-DNA interactions in related systems

a Overlays of DNA, TOPRIM domain, and WHD between *S. cerevisiae* Spo11 (cryoEM-derived monomeric model, PDB ID: 8URU) and Top6 in symmetric uncleaved (WT) and cleaved ($Top6^{(A:E342Q)}$ variant) states. **b** Overlays of the same elements between the hypothetical model of a pre-DSB Spo11 dimer by Yu et al., 2025,[15] and Top6 in symmetric uncleaved (WT) and cleaved ($Top6^{(A:E342Q)}$ variant) states. **c** Comparison of the DNA bend between an AlphaFold 3-generated model of a *M. musculus* SPO11-TOP6BL heterotetramer from Zheng et al., 2025,[16] and Top6 in symmetric uncleaved (WT) and cleaved ($Top6^{(A:E342Q)}$ mutant) states. **d** Table of protein-DNA interactions observed or predicted in Spo11 models and the WT Top6A symmetric state. The table extends the analysis carried out in Extended Data Fig. 4c (shaded in gray) from Zheng et al., 2025. Two examples of specific interaction sites are depicted in cartoon format with side chains shown as sticks and labeled. **e** TOPRIM and WH domain arrangements in Top2 (PDB ID: 4FM9) as compared to Top6 (PDB ID: 9O8P).

SUPPLEMENTARY TABLES

Supplementary Table 1: Model and map validation parameters

Data collection					
Facility	NCCAT			Johns Hopkins	
Voltage (kV)	300			300	
Magnification	130,000			130,000	
Pixel size (data) (Å)	0.96			0.93	
Total dose (e-/Å^{-2})	46			40	
Stage tilt	30° (39,964 micrographs), 0° (10,942 micrographs)			0°	
Defocus range (µm)	-0.8 to -2.5			-0.8 to -1.8	
Micrographs	50,906			26,728	
Extracted particles	4,164,804			2,237,174	
Reconstruction					
Name	WT Top6 symmetric	WT Top6 tilt-symmetric	WT Top6 lopsided	Top6$^{(A:E342Q)}$ lopsided	Top6$^{(A:E342Q)}$ cleavage
EMDB	EMD-70232	EMD-70239	EMD-70259	EMD-70206	EMD-49972
PDB	9O8P	9O8Z	9O9M	9O7O	9O0G
Final particles (no.)	201,073	176,843	293,857	370,450	329,731
Pixel size (map) (Å)	1.4385	1.4385	1.4385	0.93	0.93
Symmetry imposed	C1	C1	C1	C1	C1
Resolution (Å) (0.143 cutoff, CryoSPARC)	3.7	4.0	3.8	2.7	2.5
Local res range (Å)	3.2–8.0	3.3–8.5	3.2–17.0	2.2–15.0	2.0–10.0
cFAR / SCF*	0.13 / 0.782	0.10 / 0.781	0.03 / 0.793	0.01 / 0.509	0.47 / 0.835
Overall B-factor (Å^2)	-100.4	-106.0	-136.9	-64.0	-57.5
Model refinement and validation					
Initial models	2Q2E, 9O0G	2Q2E, 9O0G	2Q2E, 9O0G	9O0G, 2Q2E	ModelAngelo, 2Q2E
Non-hydrogen atoms	18,307	18,234	18,304	18,319	18,326
Protein / nucleotide residues	1926 / 148	1917 / 148	1926 / 148	1927 / 148	1928 / 148
Ligands	2x K, 2x Mg, 2x ANP	2x K, 3x Mg, 2x ANP	1x K, 2x Mg, 2x ANP	2x K, 2x Ca, 2x ANP	2x K, 4x Ca, 2x ANP
Mean protein ADP (Å^2)	138.18	151.03	177.35	94.25	69.08
Mean nucleotide ADP (Å^2)	146.58	197.37	266.91	169.94	100.62
Mean ligand ADP (Å^2)	115.93	131.59	151.19	86.96	62.66
RMS deviation: Bond lengths (Å)	0.004	0.005	0.007	0.007	0.004
RMS deviation: Bond angles (°)	0.773	0.952	1.159	1.075	0.743
MolProbity score	1.52	1.75	2.27	2.37	1.00
Clashscore (all atom)	3.01	2.99	7.06	6.04	1.81
Rotamer outliers (%)	0.67	1.71	3.36	5.18	0.30
Cβ outliers (%)	0.0	0.0	0.0	0.0	0.0
CaBLAM outliers (%)	3.04	5.11	4.14	3.88	1.73
Ramachandran: Favored (%)	93.53	92.24	92.23	91.81	97.71
Ramachandran: Allowed (%)	6.41	7.66	7.51	7.98	2.29
Ramachandran: Outliers (%)	0.05	0.10	0.26	0.21	0.00
Overall Q-score	0.3650	0.3220	0.2140	0.2670	0.5820
Model vs. data cross-correlation (masked) and resolution cutoff	0.84 at 4.5 Å	0.83 at 4.5 Å	0.73 at 5.0 Å	0.62 at 4.0 Å	0.90 at 2.5 Å

Supplementary Table 2: Purine (R)/pyrimidine (Y) assignment derived from the density map for the well-resolved region of mcDNA. Sites of indeterminate base assignment are yellow. The site where base assignment and minicircle pattern disagree is red. Horizontal lines mark the cleavage site on each strand. 'PTR': Phosphotyrosine.

Reference position	Assignment (R/Y)	Minicircle pattern (R/Y)		Assignment (R/Y)	Reference position
Chain H, 24 (5')	X	Y	R	X	Chain G, 51 (3')
25	Y	Y	R	R	50
26	Y	Y	R	R	49
27	Y	Y	R	R	48
28	Y	Y	R	R	47
29	R	R	Y	Y	46
30	R	R	Y	Y	45
31	Y	Y	R	R	44
32	R	R	Y	Y	43
33	Y	Y	R	R	42
34	Y	R	Y	R	41
35	R	R	Y	Y	40
36	R	R	Y	Y	39
37 (3')	Y	Y	R	R	38
38 (5'-PTR)	R	R	Y	Y	37
39	R	R	Y	Y	36 (5'-PTR)
40	R	R	Y	Y	35 (3')
41	Y	Y	R	R	34
42	Y	Y	R	R	33
43	R	R	Y	Y	32
44	Y	Y	R	R	31
45	R	R	Y	Y	30
46	Y	Y	R	R	29
47	X	R	Y	X	28
48	R	R	Y	Y	27
49	R	R	Y	Y	26
50	R	R	Y	Y	25
51	R	R	Y	Y	24
52	R	R	Y	Y	23
Chain I, 53 (3')	X	Y	R	X	Chain F, 22 (5')

Supplementary Table 3: Center-of-mass displacements and principle-axis rotations of Top6 domains relative to the Top6$^{(A:E342Q)}$ symmetric-state domain positions

	Displacement (Å)						Rotation (°)					
**Structure**	**WHD**	**TOPRIM**	**GHKL**	**H2TH**	**Transducer**	**CTD**	**WHD**	**TOPRIM**	**GHKL**	**H2TH**	**Transducer**	**CTD**
**WT Top6 symmetric**	2.65	1.31	15.78	16.04	10.83	15.04	3.05	2.91	11.13	6.63	4.49	8.19
**WT Top6 tilt-sym.**	3.64	1.42	23.29	24.17	16.54	19.78	4.79	3.03	18.3	14.36	2.31	8.8
**WT Top6 lopsided**	2.97	0.85	22.21	21.88	17.31	15.34	5.53	2.97	2.49	4.81	5.58	9.69
**Top6$^{(A:E342Q)}$ lopsided**	1.23	0.26	19.5	19.89	13.36	3.73	2.96	0.86	3.0	3.69	10.51	2.77

Supplementary Table 4: Angles of DNA deviation away from horizontal at the central kink

**Structure**	**Bend Angle (±7)**
WT Top6 symmetric	83.9°
WT Top6 tilt-symmetric	99.0°
WT Top6 lopsided	89.6°
Top6$^{(A:E342Q)}$ lopsided	101.5°
Top6$^{(A:E342Q)}$ symmetric	95.5°

SUPPLEMENTARY REFERENCES

1. Punjani, A., Rubinstein, J. L., Fleet, D. J. & Brubaker, M. A. cryoSPARC: algorithms for rapid unsupervised cryo-EM structure determination. *Nat. Methods* **14**, 290–296 (2017).
2. Punjani, A., Zhang, H. & Fleet, D. J. Non-uniform refinement: adaptive regularization improves single-particle cryo-EM reconstruction. *Nat. Methods* **17**, 1214–1221 (2020).
3. Punjani, A. & Fleet, D. J. 3D variability analysis: Resolving continuous flexibility and discrete heterogeneity from single particle cryo-EM. *J. Struct. Biol.* **213**, 107702 (2021).
4. Wagner, T. *et al.* SPHIRE-crYOLO is a fast and accurate fully automated particle picker for cryo-EM. *Commun. Biol.* **2**, 218 (2019).
5. Tan, Y. Z. *et al.* Addressing preferred specimen orientation in single-particle cryo-EM through tilting. *Nat. Methods* **14**, 793–796 (2017).
6. Baldwin, P. R. & Lyumkis, D. Non-uniformity of projection distributions attenuates resolution in Cryo-EM. *Prog. Biophys. Mol. Biol.* **150**, 160–183 (2020).
7. Cardone, G., Heymann, J. B. & Steven, A. C. One number does not fit all: Mapping local variations in resolution in cryo-EM reconstructions. *J. Struct. Biol.* **184**, 226–236 (2013).
8. Hu, X., Machius, M. & Yang, W. Monovalent cation dependence and preference of GHKL ATPases and kinases[1]. *FEBS Lett.* **544**, 268–273 (2003).
9. Nichols, M. D., DeAngelis, K., Keck, J. L. & Berger, J. M. Structure and function of an archaeal topoisomerase VI subunit with homology to the meiotic recombination factor Spo11. *EMBO J.* **18**, 6177–6188 (1999).
10. Corbett, K. D., Benedetti, P. & Berger, J. M. Holoenzyme assembly and ATP-mediated conformational dynamics of topoisomerase VI. *Nat. Struct. Mol. Biol.* **14**, 611–619 (2007).
11. Graille, M. *et al.* Crystal Structure of an Intact Type II DNA Topoisomerase: Insights into DNA Transfer Mechanisms. *Structure* **16**, 360–370 (2008).
12. Crooks, G. E., Hon, G., Chandonia, J.-M. & Brenner, S. E. WebLogo: A Sequence Logo Generator. *Genome Res.* **14**, 1188–1190 (2004).
13. Allen, A. M. B. & Maxwell, A. Phylogenetic distribution of DNA topoisomerase VI and its distinction from SPO11. *NAR Genomics Bioinforma.* **6**, lqae085 (2024).
14. Lu, X.-J. 3DNA: a software package for the analysis, rebuilding and visualization of three-dimensional nucleic acid structures. *Nucleic Acids Res.* **31**, 5108–5121 (2003).
15. Yu, Y. *et al.* Cryo-EM structures of the Spo11 core complex bound to DNA. *Nat. Struct. Mol. Biol.* **32**, 113–124 (2025).
16. Zheng, Z. *et al.* Reconstitution of SPO11-dependent double-strand break formation. *Nature* **639**, 784–791 (2025).
