# Supplementary material for: Supercoiled DNA recognition and cleavage control in topoisomerase VI
Source: Nat Commun. 2026 Feb 16;17:3092. doi: 10.1038/s41467-026-69491-0 (PMC13040014; doi:10.1038/s41467-026-69491-0)
Supplement: Supplementary file 2 — Description of Additional Supplementary Files [file 41467_2026_69491_MOESM2_ESM.pdf]

Description of Additional Supplementary Files

Supplementary Movie 1. Movement of the winged helix domain between symmetric uncleaved and cleaved states of Top6: Model morph generated in ChimeraX depicting the difference in WHD position between symmetric uncleaved (in the WT complex) and cleaved (in the Top6$^{(A:E342Q)}$ variant) states. Amino acid side chains involved in the cleavage active site are labeled, along with domains. The position of the DNA break is only meaningful in the cleaved state; its position in other conformations of the morph is an artifact of the sequence numbering, which is kept as simple as possible (by starting at 1) to avoid assumptions about the register of the DNA in uncleaved states.

Supplementary Movie 2. Comparison of five structural states imaged for Top6 bound to supercoiled DNA. Model morph generated in ChimeraX that shows conformational changes from the WT symmetric state to the tilt-symmetric state, WT lopsided state, Top6$^{(A:E342Q)}$ lopsided state, Top6$^{(A:E342Q)}$ symmetric (cleavage) state, and finally back to the WT symmetric state. Domains are colored as per Fig. 1. The unfolded region of the tilt-symmetric state transducer stalk is also missing in the other states to accommodate limitations of the morph algorithm.
