# Supplementary material for: Supercoiled DNA recognition and cleavage control in topoisomerase VI
Source: Nat Commun. 2026 Feb 16;17:3092. doi: 10.1038/s41467-026-69491-0 (PMC13040014; doi:10.1038/s41467-026-69491-0)
Supplement: Supplementary file 5 — Reporting Summary [file 41467_2026_69491_MOESM5_ESM.pdf]

nature portfolio

Corresponding author(s): James Berger

Last updated by author(s): 2025 December 30

Reporting Summary

Nature Portfolio wishes to improve the reproducibility of the work that we publish. This form provides structure for consistency and transparency in reporting. For further information on Nature Portfolio policies, see our Editorial Policies and the Editorial Policy Checklist.

Statistics

For all statistical analyses, confirm that the following items are present in the figure legend, table legend, main text, or Methods section.

n/a	Confirmed	
☐	☒	The exact sample size (n) for each experimental group/condition, given as a discrete number and unit of measurement
☐	☒	A statement on whether measurements were taken from distinct samples or whether the same sample was measured repeatedly
☒	☐	The statistical test(s) used AND whether they are one- or two-sided<br>*Only common tests should be described solely by name; describe more complex techniques in the Methods section.*
☒	☐	A description of all covariates tested
☒	☐	A description of any assumptions or corrections, such as tests of normality and adjustment for multiple comparisons
☐	☒	A full description of the statistical parameters including central tendency (e.g. means) or other basic estimates (e.g. regression coefficient) AND variation (e.g. standard deviation) or associated estimates of uncertainty (e.g. confidence intervals)
☒	☐	For null hypothesis testing, the test statistic (e.g. F, t, r) with confidence intervals, effect sizes, degrees of freedom and P value noted<br>*Give P values as exact values whenever suitable.*
☒	☐	For Bayesian analysis, information on the choice of priors and Markov chain Monte Carlo settings
☒	☐	For hierarchical and complex designs, identification of the appropriate level for tests and full reporting of outcomes
☐	☒	Estimates of effect sizes (e.g. Cohen's d, Pearson's r), indicating how they were calculated

Our web collection on statistics for biologists contains articles on many of the points above.

Software and code

Policy information about availability of computer code

Data collection	Leginon 3.6, EPU 3.6.0.6389
Data analysis	CryoSPARC v4.4.1 or v4.6.0, crYOLO v1.9.1-1.9.7, ModelAngelo v1.0.12, Coot v0.9.8.92, ChimeraX v1.9, ISOLDE v1.9

For manuscripts utilizing custom algorithms or software that are central to the research but not yet described in published literature, software must be made available to editors and reviewers. We strongly encourage code deposition in a community repository (e.g. GitHub). See the Nature Portfolio guidelines for submitting code & software for further information.

Data

Policy information about availability of data

All manuscripts must include a data availability statement. This statement should provide the following information, where applicable:

- Accession codes, unique identifiers, or web links for publicly available datasets
- A description of any restrictions on data availability
- For clinical datasets or third party data, please ensure that the statement adheres to our policy

The structural models and cryoEM density maps generated in this study have been deposited in the PDB and EMDB repositories, respectively, under the following accession codes. Wildtype symmetric state: pdb_00009o8p [http://doi.org/10.2210/pdb9o8p/pdb], EMD-70232 [https://www.ebi.ac.uk/pdbe/entry/emdb/EMD-70232]. Wildtype tilt-symmetric state: pdb_00009o8z [http://doi.org/10.2210/pdb9o8z/pdb], EMD-70239 [https://www.ebi.ac.uk/pdbe/entry/emdb/EMD-70239]. Wildtype lopsided state: pdb_00009o9m [http://doi.org/10.2210/pdb9o9m/pdb], EMD-70259 [https://www.ebi.ac.uk/pdbe/entry/emdb/

EMD-70259]. Top6(A:E342Q) lopsided state: pdb_00009o7o [http://doi.org/10.2210/pdb9o7o/pdb], EMD-70206 [https://www.ebi.ac.uk/pdbe/entry/emdb/EMD-70206]. Top6(A:E342Q) cleavage state: pdb_00009o0g [http://doi.org/10.2210/pdb9o0g/pdb], EMDB EMD-49972 [https://www.ebi.ac.uk/pdbe/entry/emdb/EMD-49972]. The cryoEM image datasets from which the maps can be derived have been deposited in the EMPIAR repository. Wildtype dataset: EMPIAR-12904 [https://doi.org/10.6019/EMPIAR-12904]. Top6(A:E342Q) dataset: EMPIAR-12914 [https://doi.org/10.6019/EMPIAR-12914]. Source Data for gels and plots are provided with this paper.

Research involving human participants, their data, or biological material

Policy information about studies with human participants or human data. See also policy information about sex, gender (identity/presentation), and sexual orientation and race, ethnicity and racism.

Reporting on sex and gender	n/a
Reporting on race, ethnicity, or other socially relevant groupings	n/a
Population characteristics	n/a
Recruitment	n/a
Ethics oversight	n/a

Note that full information on the approval of the study protocol must also be provided in the manuscript.

Field-specific reporting

Please select the one below that is the best fit for your research. If you are not sure, read the appropriate sections before making your selection.

☒ Life sciences ☐ Behavioural & social sciences ☐ Ecological, evolutionary & environmental sciences

For a reference copy of the document with all sections, see nature.com/documents/nr-reporting-summary-flat.pdf

Life sciences study design

All studies must disclose on these points even when the disclosure is negative.

Sample size	No calculation of sample size was performed. CryoEM dataset sizes were determined empirically by their ability to achieve the reported map resolutions and the availability and length of microscope sessions. Functional assays were essentially qualitative, and at least two distinct experiments were performed, as indicated in the figure captions.
Data exclusions	Maps of sufficient quality for structural modeling were reconstructed by sorting particle images according to best practices for cryoEM. Particle images of low quality or representing incomplete assemblies were removed, using standard classification algorithms, from the sets used to calculate the final maps.
Replication	Results from functional assays were reproduced successfully by performing identical experimental procedures on at least two separate occasions. CryoEM sample prep was done at least 10 times for the wildtype enzyme and at least 2 times for the cleavage-prone variant, establishing reproducibility and improvement in grid quality. Replication of data collection and analysis is generally not relevant in cryoEM, but data analyses were performed at least 2 times for each dataset to establish the robustness of particle sorting classification procedures.
Randomization	Randomization was not relevant for these structural and biochemical experiments. Particles in cryoEM or biochemical assay samples are intrinsically randomly distributed. No statistical analysis was relevant in the mutant vs wildtype gel assays.
Blinding	Blinding was not relevant for these structural and biochemical experiments. Advance knowledge of sample identity cannot influence the behavior of the sample or analytical algorithm.

Reporting for specific materials, systems and methods

We require information from authors about some types of materials, experimental systems and methods used in many studies. Here, indicate whether each material, system or method listed is relevant to your study. If you are not sure if a list item applies to your research, read the appropriate section before selecting a response.

Materials & experimental systems

n/a	Involved in the study
☒	☐ Antibodies
☒	☐ Eukaryotic cell lines
☒	☐ Palaeontology and archaeology
☒	☐ Animals and other organisms
☒	☐ Clinical data
☒	☐ Dual use research of concern
☒	☐ Plants

Methods

n/a	Involved in the study
☒	☐ ChIP-seq
☒	☐ Flow cytometry
☒	☐ MRI-based neuroimaging

Plants

Seed stocks	n/a
Novel plant genotypes	n/a
Authentication	n/a
